# Lower-Order Effects Adjustment in Quantitative Traits Model-Based Multifactor Dimensionality Reduction

**DOI:** 10.1371/journal.pone.0029594

**Published:** 2012-01-05

**Authors:** Jestinah M. Mahachie John, Tom Cattaert, François Van Lishout, Elena S. Gusareva, Kristel Van Steen

**Affiliations:** 1 Systems and Modeling Unit, Montefiore Institute, University of Liege, Liege, Belgium; 2 Bioinformatics and Modeling, GIGA-R, University of Liege, Liege, Belgium; University of North Carolina, United States of America

## Abstract

Identifying gene-gene interactions or gene-environment interactions in studies of human complex diseases remains a big challenge in genetic epidemiology. An additional challenge, often forgotten, is to account for important lower-order genetic effects. These may hamper the identification of genuine epistasis. If lower-order genetic effects contribute to the genetic variance of a trait, identified statistical interactions may simply be due to a signal boost of these effects. In this study, we restrict attention to quantitative traits and bi-allelic SNPs as genetic markers. Moreover, our interaction study focuses on 2-way SNP-SNP interactions. Via simulations, we assess the performance of different corrective measures for lower-order genetic effects in Model-Based Multifactor Dimensionality Reduction epistasis detection, using additive and co-dominant coding schemes. Performance is evaluated in terms of power and familywise error rate. Our simulations indicate that empirical power estimates are reduced with correction of lower-order effects, likewise familywise error rates. Easy-to-use automatic SNP selection procedures, SNP selection based on “top” findings, or SNP selection based on *p*-value criterion for interesting main effects result in reduced power but also almost zero false positive rates. Always accounting for main effects in the SNP-SNP pair under investigation during Model-Based Multifactor Dimensionality Reduction analysis adequately controls false positive epistasis findings. This is particularly true when adopting a co-dominant corrective coding scheme. In conclusion, automatic search procedures to identify lower-order effects to correct for during epistasis screening should be avoided. The same is true for procedures that adjust for lower-order effects prior to Model-Based Multifactor Dimensionality Reduction and involve using residuals as the new trait. We advocate using “on-the-fly” lower-order effects adjusting when screening for SNP-SNP interactions using Model-Based Multifactor Dimensionality Reduction analysis.

## Introduction

Complex diseases commonly occur in a population and are a major source of discomfort, disability and death worldwide. They are believed to arise from multiple predisposing factors, both genetic and non-genetic, each factor potentially having a modifying effect on the other. Detecting gene-gene interactions or epistasis in studies of human complex diseases is a big challenge in genetic epidemiology. An additional challenge is to account for important lower-order genetic effects in order to reduce false positive epistasis results. To date, several strategies are available, within the context of genetic association studies that specifically aim to identify and characterize gene-gene interactions. Among these strategies is the Model-Based Multifactor Dimensionality Reduction (MB-MDR) which was first introduced by Calle et al. [1]. The strategy of MB-MDR to tackle the dimensionality problem in interaction detection involves reducing a potentially high dimensional problem to a one-dimensional problem by pooling multi-locus genotypes into three groups based on association testing or modeling. Those multi-locus genotypes exhibiting some significant evidence of increasing or decreasing phenotypic mean, are labeled High group and Low group, respectively. In addition, those multi-locus genotypes that either show no evidence of association or have no sufficient sample size contribute to an additional third Model-Based Multifactor Dimensionality Reduction category, that of ‘No Evidence for association’. It has been suggested that Model-Based Multifactor Dimensionality Reduction is a useful method for identifying gene-gene interactions in case-control or family-based design for both dichotomous and quantitative traits [1], [2], . For more details on MB-MDR, we refer to the aforementioned articles. Although a power study of MB-MDR detection with and without main effects adjustment has been performed before [4], [6], these studies only involve adjusting for the known functional SNPs contributing to an epistasis effect. The preliminary results these studies gave rise to, emphasized the importance of lower-order effects adjustment when searching for gene-gene interactions and warranted a more detailed investigation.

**Figure 1:**
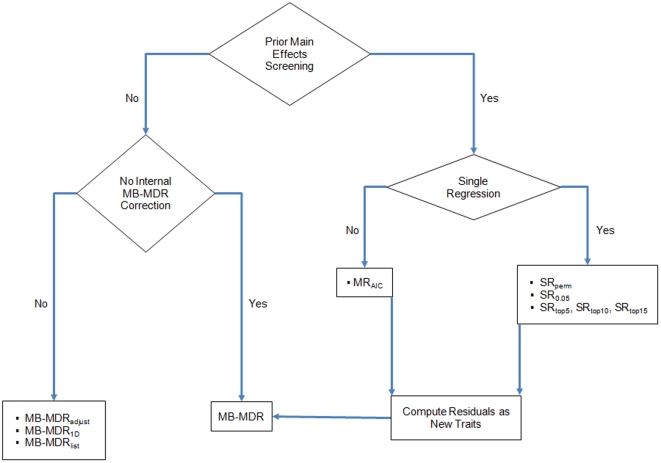
Different approaches to adjust for lower-order effects in MB-MDR epistasis screening.

In this study, we perform a thorough simulation-based investigation of the power of quantitative trait MB-MDR to identify gene-gene interactions, using different strategies to adjust for lower-order genetic effects, that may or not be part of the (functional) SNP-SNP interaction under investigation. Performance criteria used are power and familywise error rate. We perform MB-MDR epistasis analyses first without any adjustment for main effects and then with adjustments using several strategies. The proposed main effects corrections can be grouped into two categories: 1) main effects screening followed by MB-MDR applied to an adjusted trait and 2) main effect adjustment integrated in step 1 and step 2 of MB-MDR. These are depicted in Figure 1 and described in more detail in the methods section.

## Methods

### MB-MDR

**Figure 2:**
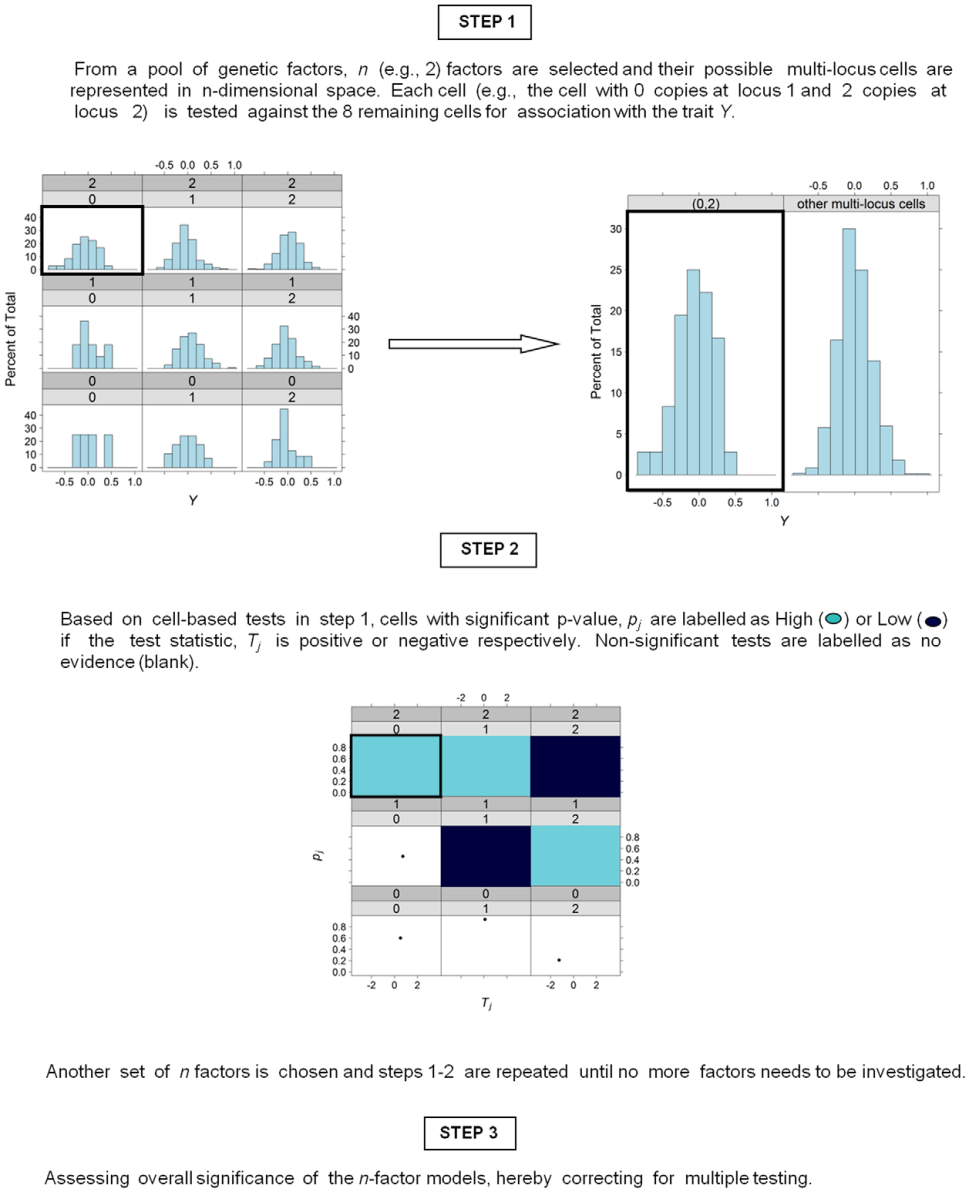
Summary of the steps involved in MB-MDR analysis.

We apply a quantitative trait MB-MDR as described in Mahachie John et al. [6] and its generalization to main effects corrections. For a sufficiently frequent bi-allelic marker, there are 3 theoretically possible genotypes. Hence, 2 bi-allelic markers give rise to 9 multi-locus cells. Each of the 9 multi-locus genotype cells alternatively constitute group 1. The remaining 8 multi-locus genotypes constitute group 2. The key MB-MDR steps are summarized in Figure 2. In MB-MDR step 1, we make use of a Student t-test at significance level 0.1 to compare the mean trait values in the 2 aforementioned groups of multi-locus genotypes. In step 2, we use the cell-based results of step 1 to label significant cells as *H*(igh) or *L*(ow) and non significant ones as ‘no evidence’, *O*. The sign of the Student's t-test statistic is used to distinguish between *H* and *L*: a positive (negative) sign refers to *H (L)*. The result is a new categorical variable with labels *H*, *L* and *O*. A new association test is then performed for the newly created construct on the trait, *Y.* In particular, we consider the maximum of Student t-tests comparing the *H*-cells versus {*L,O*}-cells and *L*-cells versus {*H,O*}-cells. In step 3, we assess the overall significance by adopting a permutation-based maxT correction [7] with 999 replicates. Although in this study we focus on 2-locus interactions, the principle of MB-MDR can be extended to single SNP-analysis (hereafter referred to as MB-MDR_1D_) and higher-order (>2) interactions (under construction).

Several methods exist to correct for lower-order effects in the context of quantitative MB-MDR epistasis screening. An overview of the considered methods in this study is given in Figure 2. A first strategy is to extensively look for potentially confounding main effects to transform the original trait to an adjusted trait and to submit this newly defined trait to MB-MDR for epistasis screening.

When correcting for main effects, a note about how to best code lower-order effects is warranted. In a GWA study, SNPs are often coded in an additive way [8]. This coding works well in practice, although power can be gained by acknowledging the true underlying genetic models [9]. For instance, if the two homozygote genotypes at a locus exhibit the same risk, different from the heterozygote risk (over-dominance), then the additive coding will have reduced power irrespective of the sample size [10]. Alternatively, several coding schemes may be investigated and a maximum statistic over screened main effects models may be selected [11]. The differing unknown operating modes of inheritance throughout the genome make it hard to flexibly and automatically acknowledge this complex inheritance spectrum.

Therefore, the route chosen in this paper, now in an epistasis context is to correct for main effects by either assuming an additive or a co-dominant coding scheme, in scenarios that involve different contributions of additive and dominance variance to main effects variance. Although some of these scenarios may be better captured by non-additive and non-co-dominant codings, the interest is in finding an all-purpose acceptable (in terms of power and type I error) way to remove the main effects signals influencing epistasis signals. Choosing between additive and co-dominant coding schemes implies choosing between the least and most severe such removal of effects.

### Main effects screening prior to MB-MDR

This screening procedure involves first adjusting for a chosen subset of main effects via parametric (linear) regression models and then considering residuals from the fitted models as a new trait for MB-MDR. For the adjustment methods involving significance assessments, we remark that whenever none of the SNPs are significant, the original trait is submitted to MB-MDR.

#### Single (univariate) regression-based searches

Important main effects can be identified via single-SNP regression models, as is done in a classical GWA setting. Hence, SNPs that meet a stringent criterion (such as governed by a Bonferroni criterion) will be labelled as “important” and are therefore good candidates to correct for in an epistasis screening. In this study, we prefer to take a less conservative route, such as a selection based on step-down maxT adjusted *p*-values with 999 replicates (Figure 1; SR_perm_). However, targeting effects standing out in a GWA main effects screening while maintaining overall type I error is quite different from targeting main effects to adjust for in an epistasis screening. Therefore, we also consider selecting “optimal” SNPs for main effects correction in the quantitative MB-MDR screening on the basis of their significance without correction for multiple testing (Figure 1; SR_0.05_) or on the basis of a ranking of the corresponding raw *p*-values (Figure 1; SR_top5_, SR_top10_, SR_top15_).

#### Multiple regression-based searches

Due to a large number of SNPs that are involved in a main effects genome-wide analysis, multiple regression-based searches are often automated. One such automated approach uses stepwise selection based on AIC (stepAIC in R package MASS, R 2.10.0). This procedure iteratively adds and/or drops variables to seek the lowest AIC score. The final model generates the list of main effects to correct for in the quantitative trait MB-MDR analysis (Figure 1; MR_AIC_).

#### Main effects adjustment as an integral part of MB-MDR

In this scenario, main effects are adjusted for “on-the-fly”, i.e. SNPs are adjusted for during the first 2 MB-MDR epistasis screening steps. Three types of adjustment are considered. A first type is to always adjust for the SNPs in the pair under investigation (Figure 1; MB-MDR_adjust_). Hence, the adjustment is done irrespective of whether a main effect is truly present. A second type is to only adjust for SNPs that are identified by MB-MDR_1D_ as significant. Here, MB-MDR_1D_ is run first and a list of genome-wide significant SNPs is identified (based on step-down maxT with 999 permutation replicates). MB-MDR epistasis screening is then performed while only adjusting for the identified SNPs for the pair under investigation (Figure 1; MB-MDR_1D_). A third type is to only adjust for significant SNPs obtained via single regression models and maxT significance assessment (Figure 1; MB-MDR_list_). Thus, for MB-MDR_1D_ and MB-MDR_list_, any of the following 3 situations can arise: a) None of the 2 SNPs is significant and no correction is performed b) One of the 2 SNPs is significant and this is adjusted for c) Both SNPS are significant and both SNPs are adjusted for.

In order to account for potentially important SNPs as an integral part of MB-MDR, we remark that the Student's t-test in MB-MDR steps 1–2 (Figure 2) is replaced by the Wald test for the interaction effect in a regression framework.

### Data Simulation

Simulated data as generated in Mahachie John et al. [6] are based on two epistasis models for SNP1 and SNP2 that incorporate varying degrees of epistasis: Model M27 and Model M170 of [12]. In order to increase the phenotypic mean, M27 requires an individual to have at least one copy of the minor allele at both loci whereas M170 requires an individual to be heterozygous at one locus and homozygous at the other. The phenotypic means for the aforementioned epistasis models only take two values, *µ_L_* (Low phenotypic mean) and *µ_H_* (High phenotypic mean). The total phenotypic variance 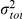, i.e. the sum of genetic variance at both loci 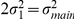 (the minor allele frequencies for the functional SNPs are taken to be the same), epistasis variance 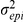, and environmental variance 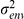, is fixed at 1. As a consequence, the total genetic variance, 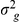 , for the two-locus model consisting of main effects variance and epistasis variance has an interpretation of a broad heritability measure. SNP1 and SNP2 have MAF equal to *p*, with *p* one of 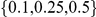. The MAFs of the other 98 markers are generated from a random uniform distribution, U(0.05,0.5). MB-MDR screening is performed on 100 SNPs in Hardy-Weinberg Equilibrium and linkage equilibrium. The total genetic variance 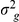 is varied as 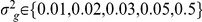. The main effects variance 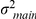 consists of additive variance 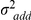 and dominance variance 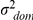. As *p* increases, the contribution to the total genetic variance of epistasis variance relative to main effects variance increases for M170 and decreases for M27, and also the contributions of additive and dominance variance to the total main effects variance change with *p* (Table 1).

**Table 1:** Theoretically derived proportions of the genetic variance due to main effects (additive and dominance) or epistasis.

Model	*p*	σ^2^_main_/σ^2^_gen_	σ^2^_add_/σ^2^_main_	σ^2^_dom_/σ^2^_main_	σ^2^_epi_/σ^2^_gen_
	0.1	0.319	0.947	0.053	0.681
M27	0.25	0.609	0.857	0.143	0.391
	0.5	0.857	0.667	0.333	0.143
	0.1	0.581	0.780	0.220	0.419
M170	0.25	0.118	0.400	0.600	0.882
	0.5	0.000	0.947	0.053	1.000

For SNP3 and SNP4 , main effects are imposed with associated variances 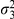 and 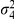, selected from a uniform distribution U(0, 0.06) such that the total main effects variance of the 4 loci (SNP1, SNP2, SNP3, SNP4) is 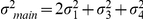. The respective modes of inheritance for SNP3 and SNP4 are additive and advantageous heterozygous. Note that SNP4 will therefore contribute to both the additive and dominance components of the main effects variance. This scenario allows us to investigate the effect of global main effects correction approaches for functional SNPs that are not part of a two-locus interaction.

In addition data are simulated under the null model for the functional pair (i.e. 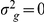) in two ways, giving rise to two null hypotheses *H_01_* and *H_02_*. *H_01_* : no genetic contribution apart from SNP3 and SNP4 as main effects and *H_02_* : no genetic contribution from any of the SNPs whatsoever.

In summary, a total of 36 simulation settings are considered. For each parameter setting, we consider 500 simulation replicates, involving 2000 unrelated individuals.

## Results

### Familywise error rates and false positive rates

Table 2 shows results for settings simulated under the null hypotheses *H_01_* and *H_02_* of no genetic associations with the trait, yet in the presence or absence of additional main effects (SNP3 and SNP4).

**Table 2:** Type I error percentages for data generated under the null hypothesis of no genetic association of the interacting pair.

Without correction and additional main effects			
*P*		Present	absent
0.1		0.982	**0.046**
0.25	**No correction**	0.984	**0.050**
0.5		0.982	**0.050**

With Correction and additional main effects					
	Way of Correction	Additive		Co-dominant	
		present	absent	present	Absent
0.1		0.676	**0.048**	**0.040**	**0.052**
0.25	MB-MDR_adjust_	0.710	**0.034**	**0.054**	**0.038**
0.5		0.740	**0.044**	**0.036**	**0.050**
0.1		0.676	**0.036**	**0.058**	**0.030**
0.25	MB-MDR_1D_	0.722	**0.040**	**0.042**	**0.036**
0.5		0.746	**0.040**	**0.038**	**0.030**
0.1		0.682	**0.038**	**0.056**	**0.030**
0.25	MB-MDR_list_	0.726	**0.036**	**0.044**	**0.032**
0.5		0.748	0.046	0.040	0.032
0.1		0.628	0.038	0.048	0.030
0.25	SR_perm_	0.660	0.036	0.058	0.030
0.5		0.678	0.046	0.044	0.032
0.1		0.576	0.014	0.006	0.010
0.25	SR_0.05_	0.604	0.006	0.012	0.000
0.5		0.636	0.022	0.008	0.008
0.1		0.552	0.008	0.000	0.002
0.25	MR_AIC_	0.578	0.004	0.002	0.000
0.5		0.616	0.012	0.000	0.006
0.1		0.582	0.014	0.008	0.008
0.25	SR_top5_	0.616	0.002	0.020	0.000
0.5		0.638	0.026	0.010	0.010
0.1		0.560	0.012	0.000	0.008
0.25	SR_top10_	0.592	0.006	0.004	0.000
0.5		0.626	0.022	0.006	0.006
0.1		0.556	0.010	0.000	0.006
0.25	SR_top15_	0.588	0.006	0.002	0.000
0.5		0.618	0.016	0.004	0.006

Results are for scenarios: with and without additional main effects (SNP3 and SNP4) contributing to the genetic variance. In bold are values within Bradley's liberal criterion of robustness.

We observe that MB-MDR type I error percentages are close to the nominal type I error rate of 5%, when no correction for main effects is performed under settings where no additional main effects act on the quantitative trait. Type I error rates are also kept under control when correction for main effects is integrated in MB-MDR epistasis screening as well as prior to MB-MDR for permutation based regression-based approach (MB-MDR_adjust_, MD-MDR_1D_, MB-MDR_list_ and SR_perm_, respectively). In particular, additive correction under *H_02_* and co-dominant correction for both *H_01_* and *H_02_*. When additional main effects are present in the data, adjusting for their effects using additive correction give rise to inflated type I error rates ranging from 55 to 74%. In contrast, when adopting a co-dominant correction, type I error is under control for MR_AIC_, and single regression-based correction methods (except SR_perm_) which are extremely conservative (Table 2: type I error rates are close to zero).

**Figure 3:**
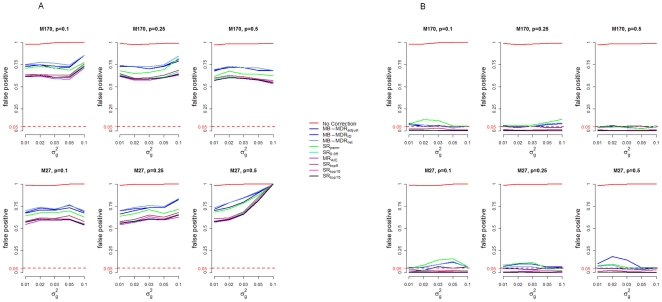
False positive percentages of MB-MDR based on additive (A) and co-dominant (B) correction. False positive percentage is defined as the proportion of simulation samples for which pairs other than the causal pair (SNP1, SNP2) are significant.

False positive rate estimates generated by MB-MDR (i.e. referring to scenarios for which one or more significantly interacting pairs other than the causal SNP pair (SNP1, SNP2)) using no correction or an additive or co-dominant correction of main effects, are shown in Figure 3. When no correction is performed, false positive rate estimates are around 100% under both M170 and M27 genetic epistasis models. In general, for additive correction false positive rate estimates range from 53 to 100% whereas for co-dominant correction, false positive rate estimates are lower and range from 0 to 19%. In particular, false positive rates for MB-MDR_adjust_ (always adjusting for main effects SNPs) in a co-dominant way range from 4 to 7%, rates that are within the interval (0.025, 0.075), satisfying Bradley's [13] liberal criterion of robustness. This criterion requires that the type I error rates are controlled for any level α of significance, if the empirical type I error rate 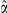 is contained in the interval 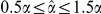. For MB-MDR_1D_ and MB-MDR_list_, false positive rates are not kept under control. The actual numerical results of the false positive profiles plotted in Figure 3 are presented in the Table S1 for M170 and Table S2 for M27. The main reason why we observe higher false positive rates under additive correction is due to the fact that SNP4 contributes to both an additive and dominance component of the main effects variance. Hence, there is also a higher chance of identifying ‘significant’ interactions for pairs involving SNP4. False positive rates are reduced when co-dominant correction is performed. Table 3 shows observed false positive rates that involve pairing with SNP3 and/or SNP4 under additive and co-dominant correction. Only MB-MDR_adjust_ (“on-the-fly” adjustment) results are shown. From Table 3, *g^2^*>0, we observe that under additive codings, false positive rates range from 51 to 61% for interactions between SNP3 and SNP4. However, for interactions with SNP3 (excluding SNP3, SNP4 interaction), false positive rates range from 0 to 6%, except for Model 27, *p* = 0.5 and *g^2^* of 0.05 and 0.1 where false positive rates are 27 and 92%, respectively. As observed in Table 1, model M27, p = 0.5 has the highest relative contribution of dominance variance, hence, additive correction does not fully account for SNP1 and SNP2.

**Table 3:** False positive percentages of MB-MDR_adjust_ involving SNP3 and/or SNP4.

			Additive			Co-dominant		
	*P*	*g^2^*	SNP3_anyotherthanSNP4	SNP3_SNP4	SNP4_anyotherthanSNP3	SNP3_anyotherthanSNP4	SNP3_SNP4	SNP4_anyotherthanSNP3
	0.1		0.002	0.520	0.660	0.000	0.000	0.000
*H_01_*	0.25	0	0.000	0.556	0.688	0.000	0.000	0.000
	0.5		0.002	0.608	0.722	0.004	0.000	0.002
		0.01	0.002	0.584	0.704	0.004	0.000	0.000
		0.02	0.008	0.582	0.724	0.002	0.000	0.000
	0.1	0.03	0.000	0.572	0.690	0.000	0.000	0.000
		0.05	0.008	0.534	0.676	0.002	0.000	0.000
		0.1	0.072	0.540	0.752	0.000	0.000	0.000
		0.01	0.002	0.598	0.714	0.000	0.000	0.004
		0.02	0.000	0.558	0.712	0.002	0.000	0.002
M170	0.25	0.03	0.000	0.544	0.686	0.000	0.000	0.000
		0.05	0.004	0.536	0.706	0.002	0.000	0.000
		0.1	0.032	0.566	0.738	0.000	0.000	0.000
		0.01	0.000	0.526	0.664	0.000	0.000	0.002
		0.02	0.000	0.588	0.708	0.000	0.000	0.002
	0.5	0.03	0.002	0.544	0.692	0.002	0.000	0.002
		0.05	0.002	0.550	0.666	0.000	0.000	0.000
		0.1	0.002	0.528	0.662	0.002	0.000	0.000
		0.01	0.002	0.532	0.662	0.000	0.000	0.000
		0.02	0.000	0.564	0.690	0.000	0.000	0.000
	0.1	0.03	0.000	0.554	0.680	0.002	0.000	0.000
		0.05	0.000	0.562	0.704	0.002	0.000	0.000
		0.1	0.000	0.518	0.638	0.000	0.000	0.000
		0.01	0.002	0.512	0.652	0.000	0.000	0.002
		0.02	0.004	0.520	0.682	0.004	0.000	0.000
M27	0.25	0.03	0.000	0.562	0.700	0.002	0.000	0.000
		0.05	0.000	0.546	0.700	0.000	0.000	0.002
		0.1	0.042	0.564	0.734	0.002	0.000	0.000
		0.01	0.000	0.546	0.672	0.000	0.000	0.002
		0.02	0.020	0.508	0.684	0.000	0.000	0.000
	0.5	0.03	0.060	0.518	0.706	0.000	0.000	0.002
		0.05	0.272	0.536	0.806	0.000	0.000	0.000
		0.1	0.912	0.590	0.974	0.000	0.000	0.000

False positive percentages shown are for identifying interaction between SNP3 and SNP4 and for interactions between SNP3 or SNP4 and at least one other SNP for null data scenario under *H_01_* and for models M170 and M27.

### Empirical power estimates

**Figure 4:**
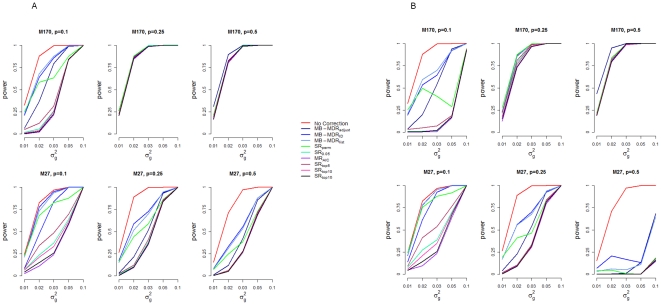
Power to identify SNP1, SNP2, as significant for additive (A) and co-dominant (B) correction.

Power profiles of MB-MDR to detect the correct interacting pair (SNP1, SNP2) without and with different ways of adjustment of main effects are shown in Figure 4. Empirical power estimates are presented as Table S1 for M170 and Table S2 for M27. In this section, we focus on scenarios where there is some remarkable degree of main effects contributing to the genetic variance (M170: *p* = 0.1, M27: *p* = 0.25 and 0.5). For a detailed view on variance decomposition into main and epistatic effects, we refer to [6]. Under the aforementioned scenarios, the profile for no correction always has the highest power. Under M170, the empirical power estimates for this profile range from 33 to 100% for *p* = 0.1. Under M27, the power estimates range from 27 to 100% and from 15 to 100%, for *p* = 0.25 and 0.5 respectively. Irrespective of whether main effects are corrected for using additive or co-dominant coding, profiles for the considered multiple-regression, MR_AIC_ and single regression-based methods that do not involve multiple testing (SR_0.05_, SR_top5_, SR_top10_ and SR_top15_) tend to follow the same trajectory, giving rise to the lowest empirical power estimates. With additive adjustments, empirical power estimates for these corrective ways range from 0 to 100% for both models M27 and M170. With co-dominant adjustments, power estimates range from 0 to 93% , for M170, *p* = 0.1, from 0 to 100% and from 0 to 18%, for model M27, *p* = 0.25 and *p* = 0.5 respectively. Estimates for MB-MDR_adjust_ (corrective methods that are integrated as part of MB-MDR) , range from 6 to 100% for M170, *p* = 0.1, from 3 to 100% for M27 with *p* = 0.25 and from 1 to 100% for M27 with *p* = 0.5, when additive corrections are performed. Under co-dominant corrections the estimates range from 4 to 100% for M170 (*p* = 0.1) and from 4 to 100% or from 0 to 68% for M27 (*p* = 0.25 and *p* = 0.5 respectively).

## Discussion

The identification of genetic susceptibility loci for human complex diseases has been rather successful due to the ability to combine different genome-wide association studies via meta-analyses. In the quest for the missing heritability, genome-wide association interaction studies have become increasingly popular and the field shows a boost in methodological developments [14]. When lower-order effects are not appropriately accounted for in epistasis screening, derived results may not be trustworthy and conclusions about genuine epistasis may be ungrounded.

Indeed, the challenge is to find epistasis effects above and beyond singular marker contributing effects, should there be any. In this work, we investigated the power of MB-MDR for quantitative traits and unrelated individuals, while targeting gene–gene interactions accounting for potential main effects.

As was already observed in [6], MB-MDR adequately controls type I rate at 5% when no association is present (null data). Under additive corrections, type I error and false positive rates are high irrespective of the adjustment method considered but controlled under co-dominant corrections. This is due to the existence of SNP4, which was simulated with both additive and dominance effects (advantageous heterozygous). Hence, additive adjustment does not fully remove the effect of SNP4. As shown before in Table 3, the consequence is that a number of SNPs appear to be significantly interacting with SNP4. Not surprisingly, this occurs more often under additive correction compared to co-dominant correction. This is because when we correct for main effects using the co-dominant model, we remove all the effect of SNP4, and hence false positive results are only by chance (5% nominal error rate). When no main effects adjustment is implemented, MB-MDR gives even higher false positive rate rates.

Lower power profiles under co-dominant corrections in Figure 4 are explained by the different contributions of additive and dominance effects to the total main effects variance as already shown in Table 1. When there is a remarkable contribution of dominance effect, as mentioned before, additive coding does not fully remove main effect contribution of the interacting SNPs. For instance, under M27, when the contribution of main effects is maximum (*p* = 0.5), almost 33% of the main effects variance is dominance, hence a huge difference in the power profiles between additive and co-dominant codings.

Interestingly, easy-to-use automatic subset selection procedures (MR_AIC_) and single regression-based identification of important main effects prior to MB-MDR screening result in lower power and almost zero false positive rates. Often, a list of top SNPs is generated to derive disease genetic risk scores. Some of these SNPs may reach user-defined significance, some may even reach genome-wide significance and some may not be significant at all. Hence, correcting for SNPs in such a list (e.g. top5, 10, 15) may remove more of the trait's variability than is really necessary, especially when correction for multiple testing is not performed. Note that we considered a minimum of 5 top findings since at least 4 SNPs were allowed to contribute to the main effects variance.

In order to attain sufficient power, any main effects corrective method that leads to an over-correction during epistasis screening should be avoided. All considered residual-based approaches (MRAIC, SR0.05, SRperm, SRtop5, SRtop10, SRtop15) led to uncontrolled false positive rates. This can be explained by either the way the residuals were obtained (inappropriate main effects coding) or by the non-exhaustive list of markers considered in the residual computation.

Only co-dominantly correcting for significant SNPs as integral part of MB-MDR screening perform much better. However, the poor performance of MB-MDR_1D_ and MB-MDR_list_ and the excellent performance of MB-MDR_adjust_ in terms of controlling false positive epistasis rates supports the intuition that it (only) matters to correct for those SNPs that are involved in the SNP pair under investigation, when no other SNPs are expected to modify the effect of that pair.

The aforementioned discussion clearly raises questions about how to best correct for lower-order effects when higher-order (>2) interactions are targeted. In either case, to aid in interpretation of results, it is always a good practice to assess the joint information of clusters of SNPs that contribute to the trait variability [15].

Finally, we emphasize that most statistical epistasis detection methods can be decomposed into a core component and a multiple testing correction component. Keeping the core component, but using a more refined multiple testing correction can generally enhance its performance. For instance, assumptions underlying the maxT procedure of [7] that is implemented in MB-MDR are likely to be violated for MB-MDR_1D_ and MB-MDR_list_ . Indeed, the null and the alternative hypotheses per pair of SNPs under investigation are no longer the same for all interaction tests.

In conclusion, rather than adjusting for lower-order effects prior to MB-MDR and using residuals as the new trait, or adjusting only for significant SNP(s), we advocate an “on-the-fly” main effects adjustment (MB-MDR_adjust_). This type of adjustment only removes potential main effects contributions in the pair under investigation but keeps the null and alternative hypotheses similar from one pair of SNPs to another. We have shown that the commonly used additive coding in the “on-the-fly” adjustment (MB-MDR_adjust_) is not sufficient and leads to overly optimistic results and that co-dominant adjustments are to be preferred. This will ensure an acceptable balance between type I error and power to identify the interactions.

Realistic settings often involve both additive and dominance genetic effects to the trait under investigation. Equivalent to our co-dominant coding, a perhaps biologically more meaningful coding involves introducing 2 variables *X_1_* and *X_2_* with values −1, 0, 1 and −1/2, 1/2, −1/2, respectively, for homogenous wild type, heterozygote and homozygote mutant genotypes. In such a coding scheme, both additive and dominant scales are represented. This 2-parameter coding is statistically attractive since it is invariant to allele coding (i.e. whether coding homogenous wild type as 1 or homozygote mutant genotypes as 1 for *X_1_*) [16]. The utility of the aforementioned coding as a way to adjust for lower-order effects in MB-MDR higher-order epistasis screening will be the subject of future research.

### Software

The MB-MDR software with the MB-MDR_adjust_ option is available upon request from the first author (jmahachieulg.ac.be).

## Supporting Information

Table S1 **MB-MDR power and false positives under the epistasis model M170.** False positive percentage is defined as the proportion of simulation samples for which at least one pair other than the causal pair (SNP1, SNP2) are significant. Power is defined as the proportion of simulated samples of which the causal pair (SNP1, SNP2) is significant. Results are for correction of main effects and for different ways of main effect correction. In bold are values within Bradley's liberal criterion of robustness.
(DOC)

Table S2 **MB-MDR power and false positives under the epistasis model M27.** False positive percentage is defined as the proportion of simulation samples for which at least one pair other than the causal pair (SNP1, SNP2) are significant. Power is defined as the proportion of simulated samples of which the causal pair (SNP1, SNP2) is significant. Results are for correction of main effects and for different ways of main effect correction. In bold are values within Bradley's liberal criterion of robustness.
(DOC)
