# Supplementary material for: Lower-Order Effects Adjustment in Quantitative Traits Model-Based Multifactor Dimensionality Reduction
Source: PLoS One. 2012 Jan 5;7(1):e29594. doi: 10.1371/journal.pone.0029594 (PMC3252336; doi:10.1371/journal.pone.0029594)
Supplement: Table S1 — MB-MDR power and false positives under the epistasis model M170. False positive percentage is defined as the proportion of simulation samples for which at least one pair other than the causal pair (SNP1, SNP2) are significant. Power is defined as the proportion of simulated samples of which the causal pair (SNP1, SNP2) is significant. Results are for correction of main effects and for different ways of main effect correction. In bold are values within Bradley's liberal criterion of robustness. (DOC) [file pone.0029594.s001.doc]

**Table S1.** MB-MDR power and false positives under the epistasis model M170

Legend: False positive percentage is defined as the proportion of simulation samples for which at least one pair other than the causal pair (SNP1, SNP2) are significant. Power is defined as the proportion of simulated samples of which the causal pair (SNP1, SNP2) is significant. Results are for correction of main effects and for different ways of main effect correction. In bold are values within Bradley’s liberal criterion of robustness

|  |  | Power | | | | False Positives | | |
| --- | --- | --- | --- | --- | --- | --- | --- | --- |
| p | g^2^ | No  Correction | Main Effects Correction | Additive | Co-dominant | No  Correction | Additive | Co-dominant |
|  |  |  | MB-MDR_adjust_ | 0.064 | 0.036 |  | 0.726 | **0.074** |
|  |  |  | MB-MDR_1D_ | 0.210 | 0.194 |  | 0.744 | 0.092 |
|  |  |  | MB-MDR_list_ | 0.226 | 0.208 |  | 0.752 | 0.084 |
|  | 0.01 | 0.326 | SR_perm_ | 0.252 | 0.252 | 0.978 | 0.708 | 0.078 |
|  |  |  | SR_0.05_ | 0.020 | 0.008 |  | 0.624 | 0.016 |
|  |  |  | MR_AIC_ | 0.002 | 0.000 |  | 0.612 | 0.006 |
|  |  |  | SR_top5_ | 0.050 | 0.034 |  | 0.638 | **0.026** |
|  |  |  | MB-MDR_adjust_ | 0.368 | 0.202 |  | 0.746 | **0.050** |
|  |  |  | MB-MDR_1D_ | 0.642 | 0.534 |  | 0.748 | **0.052** |
|  |  |  | MB-MDR_list_ | 0.678 | 0.594 |  | 0.776 | **0.068** |
|  | 0.02 | 0.878 | SR_perm_ | 0.582 | 0.496 | 0.982 | 0.728 | 0.128 |
|  |  |  | SR_0.05_ | 0.060 | 0.014 |  | 0.640 | 0.012 |
|  |  |  | MR_AIC_ | 0.024 | 0.004 |  | 0.620 | 0.004 |
|  |  |  | SR_top5_ | 0.122 | 0.050 |  | 0.638 | 0.022 |
|  |  |  | MB-MDR_adjust_ | 0.794 | 0.540 |  | 0.708 | **0.060** |
|  |  |  | MB-MDR_1D_ | 0.856 | 0.644 |  | 0.734 | **0.064** |
|  |  |  | MB-MDR_list_ | 0.874 | 0.696 |  | 0.766 | **0.060** |
| 0.1 | 0.03 | 0.996 | SR_perm_ | 0.632 | 0.406 | 0.996 | 0.712 | 0.120 |
|  |  |  | SR_0.05_ | 0.260 | 0.018 |  | 0.606 | 0.006 |
|  |  |  | MR_AIC_ | 0.216 | 0.010 |  | 0.592 | 0.004 |
|  |  |  | SR_top5_ | 0.314 | 0.072 |  | 0.630 | **0.034** |
|  |  |  | MB-MDR_adjust_ | 0.990 | 0.940 |  | 0.718 | **0.050** |
|  |  |  | MB-MDR_1D_ | 0.992 | 0.922 |  | 0.724 | **0.034** |
|  |  |  | MB-MDR_list_ | 0.994 | 0.924 |  | 0.742 | **0.036** |
|  | 0.05 | 1.000 | SR_perm_ | 0.874 | 0.290 | 1.000 | 0.686 | **0.058** |
|  |  |  | SR_0.05_ | 0.840 | 0.176 |  | 0.618 | 0.006 |
|  |  |  | MR_AIC_ | 0.842 | 0.166 |  | 0.582 | 0.002 |
|  |  |  | SR_top5_ | 0.842 | 0.190 |  | 0.630 | 0.014 |
|  |  |  | MB-MDR_adjust_ | 1.000 | 1.000 |  | 0.854 | **0.054** |
|  |  |  | MB-MDR_1D_ | 1.000 | 1.000 |  | 0.854 | **0.060** |
|  |  |  | MB-MDR_list_ | 1.000 | 1.000 |  | 0.852 | **0.050** |
|  | 0.1 | 1.000 | SR_perm_ | 1.000 | 0.942 | 1.000 | 0.772 | **0.054** |
|  |  |  | SR_0.05_ | 1.000 | 0.930 |  | 0.740 | 0.008 |
|  |  |  | MR_AIC_ | 1.000 | 0.922 |  | 0.710 | 0.000 |
|  |  |  | SR_top5_ | 1.000 | 0.938 |  | 0.756 | 0.016 |

**Table S1 Continued**

|  |  | Power | | | | False Positives | | |
| --- | --- | --- | --- | --- | --- | --- | --- | --- |
| p | g^2^ | No  Correction | Main Effects Correction | Additive | Co-dominant | No  Correction | Additive | Co-dominant |
|  |  |  | MB-MDR_adjust_ | 0.264 | 0.234 |  | 0.730 | **0.050** |
|  |  |  | MB-MDR_1D_ | 0.234 | 0.232 |  | 0.740 | **0.064** |
|  |  |  | MB-MDR_list_ | 0.234 | 0.230 |  | 0.736 | **0.056** |
|  | 0.01 | 0.234 | SR_perm_ | 0.262 | 0.280 | 0.988 | 0.680 | **0.058** |
|  |  |  | SR_0.05_ | 0.228 | 0.214 |  | 0.638 | 0.018 |
|  |  |  | MR_AIC_ | 0.208 | 0.126 |  | 0.614 | 0.002 |
|  |  |  | SR_top5_ | 0.234 | 0.226 |  | 0.648 | 0.020 |
|  |  |  | MB-MDR_adjust_ | 0.872 | 0.862 |  | 0.724 | **0.040** |
|  |  |  | MB-MDR_1D_ | 0.862 | 0.856 |  | 0.726 | **0.050** |
|  |  |  | MB-MDR_list_ | 0.862 | 0.860 |  | 0.730 | **0.048** |
|  | 0.02 | 0.864 | SR_perm_ | 0.880 | 0.874 | 0.974 | 0.648 | **0.058** |
|  |  |  | SR_0.05_ | 0.858 | 0.786 |  | 0.588 | 0.004 |
|  |  |  | MR_AIC_ | 0.842 | 0.728 |  | 0.574 | 0.006 |
|  |  |  | SR_top5_ | 0.864 | 0.812 |  | 0.596 | 0.010 |
|  |  |  | MB-MDR_adjust_ | 0.996 | 0.996 |  | 0.700 | **0.054** |
|  |  |  | MB-MDR_1D_ | 0.996 | 0.996 |  | 0.700 | **0.050** |
|  |  |  | MB-MDR_list_ | 0.996 | 0.996 |  | 0.724 | **0.054** |
| 0.25 | 0.03 | 0.996 | SR_perm_ | 0.996 | 0.994 | 0.982 | 0.662 | **0.070** |
|  |  |  | SR_0.05_ | 0.994 | 0.982 |  | 0.590 | 0.006 |
|  |  |  | MR_AIC_ | 0.986 | 0.964 |  | 0.572 | 0.000 |
|  |  |  | SR_top5_ | 0.990 | 0.988 |  | 0.602 | 0.012 |
|  |  |  | MB-MDR_adjust_ | 1.000 | 1.000 |  | 0.728 | **0.040** |
|  |  |  | MB-MDR_1D_ | 1.000 | 1.000 |  | 0.732 | **0.074** |
|  |  |  | MB-MDR_list_ | 1.000 | 1.000 |  | 0.746 | 0.082 |
|  | 0.05 | 1.000 | SR_perm_ | 1.000 | 1.000 | 0.990 | 0.694 | 0.096 |
|  |  |  | SR_0.05_ | 0.996 | 0.998 |  | 0.608 | 0.010 |
|  |  |  | MR_AIC_ | 1.000 | 1.000 |  | 0.602 | 0.006 |
|  |  |  | SR_top5_ | 1.000 | 1.000 |  | 0.630 | 0.016 |
|  |  |  | MB-MDR_adjust_ | 1.000 | 1.000 |  | 0.788 | **0.038** |
|  |  |  | MB-MDR_1D_ | 1.000 | 1.000 |  | 0.806 | 0.082 |
|  |  |  | MB-MDR_list_ | 1.000 | 1.000 |  | 0.846 | 0.092 |
|  | 0.1 | 1.000 | SR_perm_ | 1.000 | 1.000 | 0.992 | 0.820 | 0.132 |
|  |  |  | SR_0.05_ | 1.000 | 1.000 |  | 0.668 | 0.006 |
|  |  |  | MR_AIC_ | 1.000 | 1.000 |  | 0.630 | 0.004 |
|  |  |  | SR_top5_ | 1.000 | 1.000 |  | 0.688 | 0.016 |

**Table S1 Continued**

|  |  | Power | | | | False Positives | | |
| --- | --- | --- | --- | --- | --- | --- | --- | --- |
| p | g^2^ | No  Correction | Main Effects Correction | Additive | Co-dominant | No  Correction | Additive | Co-dominant |
|  |  |  | MB-MDR_adjust_ | 0.312 | 0.436 |  | 0.686 | **0.044** |
|  |  |  | MB-MDR_1D_ | 0.196 | 0.192 |  | 0.676 | **0.044** |
|  |  |  | MB-MDR_list_ | 0.194 | 0.192 |  | 0.692 | **0.040** |
|  | 0.01 | 0.196 | SR_perm_ | 0.214 | 0.220 | 0.972 | 0.616 | **0.036** |
|  |  |  | SR_0.05_ | 0.196 | 0.206 |  | 0.588 | 0.014 |
|  |  |  | MR_AIC_ | 0.166 | 0.194 |  | 0.568 | 0.006 |
|  |  |  | SR_top5_ | 0.198 | 0.206 |  | 0.598 | 0.016 |
|  |  |  | MB-MDR_adjust_ | 0.896 | 0.948 |  | 0.716 | **0.048** |
|  |  |  | MB-MDR_1D_ | 0.804 | 0.802 |  | 0.728 | **0.062** |
|  |  |  | MB-MDR_list_ | 0.804 | 0.802 |  | 0.730 | **0.054** |
|  | 0.02 | 0.806 | SR_perm_ | 0.830 | 0.840 | 0.984 | 0.676 | **0.044** |
|  |  |  | SR_0.05_ | 0.826 | 0.818 |  | 0.616 | 0.004 |
|  |  |  | MR_AIC_ | 0.804 | 0.794 |  | 0.604 | 0.000 |
|  |  |  | SR_top5_ | 0.828 | 0.826 |  | 0.626 | 0.006 |
|  |  |  | MB-MDR_adjust_ | 0.998 | 1.000 |  | 0.716 | **0.054** |
|  |  |  | MB-MDR_1D_ | 0.992 | 0.992 |  | 0.714 | **0.040** |
|  |  |  | MB-MDR_list_ | 0.992 | 0.992 |  | 0.710 | **0.036** |
| 0.5 | 0.03 | 0.992 | SR_perm_ | 0.992 | 0.992 | 0.980 | 0.640 | **0.048** |
|  |  |  | SR_0.05_ | 0.990 | 0.988 |  | 0.596 | 0.010 |
|  |  |  | MR_AIC_ | 0.988 | 0.986 |  | 0.592 | 0.006 |
|  |  |  | SR_top5_ | 0.990 | 0.990 |  | 0.612 | 0.010 |
|  |  |  | MB-MDR_adjust_ | 1.000 | 1.000 |  | 0.682 | **0.054** |
|  |  |  | MB-MDR_1D_ | 1.000 | 1.000 |  | 0.684 | **0.030** |
|  |  |  | MB-MDR_list_ | 1.000 | 1.000 |  | 0.710 | **0.030** |
|  | 0.05 | 1.000 | SR_perm_ | 1.000 | 1.000 | 0.984 | 0.640 | **0.026** |
|  |  |  | SR_0.05_ | 0.998 | 0.998 |  | 0.584 | 0.004 |
|  |  |  | MR_AIC_ | 1.000 | 1.000 |  | 0.572 | 0.000 |
|  |  |  | SR_top5_ | 1.000 | 1.000 |  | 0.586 | 0.010 |
|  |  |  | MB-MDR_adjust_ | 1.000 | 1.000 |  | 0.686 | **0.040** |
|  |  |  | MB-MDR_1D_ | 1.000 | 1.000 |  | 0.680 | **0.044** |
|  |  |  | MB-MDR_list_ | 1.000 | 1.000 |  | 0.684 | **0.044** |
|  | 0.1 | 1.000 | SR_perm_ | 1.000 | 1.000 | 0.986 | 0.624 | **0.062** |
|  |  |  | SR_0.05_ | 1.000 | 1.000 |  | 0.570 | 0.008 |
|  |  |  | MR_AIC_ | 1.000 | 1.000 |  | 0.534 | 0.000 |
|  |  |  | SR_top5_ | 1.000 | 1.000 |  | 0.568 | 0.016 |
