# Supplementary material for: Lower-Order Effects Adjustment in Quantitative Traits Model-Based Multifactor Dimensionality Reduction
Source: PLoS One. 2012 Jan 5;7(1):e29594. doi: 10.1371/journal.pone.0029594 (PMC3252336; doi:10.1371/journal.pone.0029594)
Supplement: Table S2 — MB-MDR power and false positives under the epistasis model M27. False positive percentage is defined as the proportion of simulation samples for which at least one pair other than the causal pair (SNP1, SNP2) are significant. Power is defined as the proportion of simulated samples of which the causal pair (SNP1, SNP2) is significant. Results are for correction of main effects and for different ways of main effect correction. In bold are values within Bradley's liberal criterion of robustness. (DOC) [file pone.0029594.s002.doc]

**Table S2.** MB-MDR power and false positives under the epistasis model M27

|  |  | Power | | | | False Positives | | |
| --- | --- | --- | --- | --- | --- | --- | --- | --- |
| p | g^2^ | No  Correction | Main Effects Correction | Additive | Co-dominant | No  Correction | Additive | Co-dominant |
|  |  |  | MB-MDR_adjust_ | 0.088 | 0.140 |  | 0.672 | **0.032** |
|  |  |  | MB-MDR_1D_ | 0.210 | 0.210 |  | 0.680 | **0.058** |
|  |  |  | MB-MDR_list_ | 0.212 | 0.216 |  | 0.700 | **0.050** |
|  | 0.01 | 0.242 | SR_perm_ | 0.228 | 0.244 | 0.988 | 0.640 | **0.048** |
|  |  |  | SR_0.05_ | 0.070 | 0.078 |  | 0.574 | 0.008 |
|  |  |  | MR_AIC_ | 0.026 | 0.046 |  | 0.546 | 0.004 |
|  |  |  | SR_top5_ | 0.084 | 0.094 |  | 0.580 | 0.010 |
|  |  |  | MB-MDR_adjust_ | 0.472 | 0.616 |  | 0.708 | **0.028** |
|  |  |  | MB-MDR_1D_ | 0.774 | 0.778 |  | 0.728 | **0.062** |
|  |  |  | MB-MDR_list_ | 0.732 | 0.782 |  | 0.740 | **0.066** |
|  | 0.02 | 0.826 | SR_perm_ | 0.680 | 0.754 | 0.98 | 0.676 | 0.086 |
|  |  |  | SR_0.05_ | 0.236 | 0.274 |  | 0.618 | 0.018 |
|  |  |  | MR_AIC_ | 0.110 | 0.096 |  | 0.592 | 0.002 |
|  |  |  | SR_top5_ | 0.344 | 0.414 |  | 0.620 | **0.030** |
|  |  |  | MB-MDR_adjust_ | 0.834 | 0.926 |  | 0.706 | **0.070** |
|  |  |  | MB-MDR_1D_ | 0.948 | 0.952 |  | 0.712 | 0.094 |
|  |  |  | MB-MDR_list_ | 0.936 | 0.952 |  | 0.718 | 0.094 |
| 0.1 | 0.03 | 0.968 | SR_perm_ | 0.824 | 0.880 | 0.984 | 0.676 | 0.142 |
|  |  |  | SR_0.05_ | 0.370 | 0.388 |  | 0.612 | 0.006 |
|  |  |  | MR_AIC_ | 0.238 | 0.236 |  | 0.590 | 0.000 |
|  |  |  | SR_top5_ | 0.484 | 0.538 |  | 0.612 | 0.018 |
|  |  |  | MB-MDR_adjust_ | 0.998 | 1.000 |  | 0.732 | **0.052** |
|  |  |  | MB-MDR_1D_ | 0.994 | 0.994 |  | 0.766 | 0.120 |
|  |  |  | MB-MDR_list_ | 0.996 | 0.994 |  | 0.756 | 0.126 |
|  | 0.05 | 1.000 | SR_perm_ | 0.876 | 0.916 | 0.998 | 0.698 | 0.156 |
|  |  |  | SR_0.05_ | 0.636 | 0.702 |  | 0.600 | 0.012 |
|  |  |  | MR_AIC_ | 0.572 | 0.638 |  | 0.598 | 0.002 |
|  |  |  | SR_top5_ | 0.692 | 0.766 |  | 0.644 | **0.026** |
|  |  |  | MB-MDR_adjust_ | 1.000 | 1.000 |  | 0.672 | **0.066** |
|  |  |  | MB-MDR_1D_ | 1.000 | 1.000 |  | 0.688 | **0.064** |
|  |  |  | MB-MDR_list_ | 1.000 | 1.000 |  | 0.700 | **0.062** |
|  | 0.1 | 1.000 | SR_perm_ | 1.000 | 1.000 | 1.000 | 0.628 | **0.068** |
|  |  |  | SR_0.05_ | 1.000 | 1.000 |  | 0.556 | 0.010 |
|  |  |  | MR_AIC_ | 1.000 | 1.000 |  | 0.536 | 0.002 |
|  |  |  | SR_top5_ | 1.000 | 1.000 |  | 0.576 | 0.020 |

Legend: False positive percentage is defined as the proportion of simulation samples for which at least one pair other than the causal pair (SNP1, SNP2) are significant. Power is defined as the proportion of simulated samples of which the causal pair (SNP1, SNP2) is significant. Results are for correction of main effects and for different ways of main effect correction. In bold are values within Bradley’s liberal criterion of robustness

**Table S2 Continued**

|  |  | Power | | | | False Positives | | |
| --- | --- | --- | --- | --- | --- | --- | --- | --- |
| p | g^2^ | No  Correction | Main Effects Correction | Additive | Co-dominant | No  Correction | Additive | Co-dominant |
|  |  |  | MB-MDR_adjust_ | 0.032 | 0.042 |  | 0.662 | **0.048** |
|  |  |  | MB-MDR_1D_ | 0.176 | 0.168 |  | 0.696 | **0.074** |
|  |  |  | MB-MDR_list_ | 0.152 | 0.170 |  | 0.700 | **0.062** |
|  | 0.01 | 0.266 | SR_perm_ | 0.160 | 0.192 | 0.986 | 0.636 | 0.086 |
|  |  |  | SR_0.05_ | 0.018 | 0.016 |  | 0.566 | 0.004 |
|  |  |  | MR_AIC_ | 0.006 | 0.008 |  | 0.542 | 0.004 |
|  |  |  | SR_top5_ | 0.026 | 0.018 |  | 0.578 | 0.008 |
|  |  |  | MB-MDR_adjust_ | 0.216 | 0.230 |  | 0.702 | **0.044** |
|  |  |  | MB-MDR_1D_ | 0.588 | 0.558 |  | 0.722 | 0.096 |
|  |  |  | MB-MDR_list_ | 0.516 | 0.554 |  | 0.738 | **0.068** |
|  | 0.02 | 0.890 | SR_perm_ | 0.440 | 0.410 | 0.998 | 0.666 | 0.106 |
|  |  |  | SR_0.05_ | 0.106 | 0.094 |  | 0.582 | 0.010 |
|  |  |  | MR_AIC_ | 0.094 | 0.096 |  | 0.570 | 0.010 |
|  |  |  | SR_top5_ | 0.124 | 0.104 |  | 0.602 | 0.016 |
|  |  |  | MB-MDR_adjust_ | 0.514 | 0.538 |  | 0.734 | **0.032** |
|  |  |  | MB-MDR_1D_ | 0.724 | 0.700 |  | 0.732 | 0.098 |
|  |  |  | MB-MDR_list_ | 0.698 | 0.676 |  | 0.756 | **0.064** |
| 0.25 | 0.03 | 0.996 | SR_perm_ | 0.588 | 0.464 | 0.994 | 0.710 | 0.116 |
|  |  |  | SR_0.05_ | 0.380 | 0.336 |  | 0.630 | 0.010 |
|  |  |  | MR_AIC_ | 0.354 | 0.304 |  | 0.602 | 0.002 |
|  |  |  | SR_top5_ | 0.410 | 0.330 |  | 0.650 | 0.018 |
|  |  |  | MB-MDR_adjust_ | 0.930 | 0.934 |  | 0.732 | **0.056** |
|  |  |  | MB-MDR_1D_ | 0.938 | 0.926 |  | 0.734 | **0.064** |
|  |  |  | MB-MDR_list_ | 0.944 | 0.930 |  | 0.746 | **0.042** |
|  | 0.05 | 1.000 | SR_perm_ | 0.862 | 0.838 | 1.000 | 0.668 | **0.052** |
|  |  |  | SR_0.05_ | 0.840 | 0.822 |  | 0.608 | 0.004 |
|  |  |  | MR_AIC_ | 0.836 | 0.796 |  | 0.596 | 0.000 |
|  |  |  | SR_top5_ | 0.854 | 0.834 |  | 0.628 | 0.022 |
|  |  |  | MB-MDR_adjust_ | 1.000 | 1.000 |  | 0.826 | **0.062** |
|  |  |  | MB-MDR_1D_ | 1.000 | 1.000 |  | 0.834 | **0.064** |
|  |  |  | MB-MDR_list_ | 1.000 | 1.000 |  | 0.836 | **0.046** |
|  | 0.1 | 1.000 | SR_perm_ | 1.000 | 1.000 | 1.000 | 0.716 | **0.046** |
|  |  |  | SR_0.05_ | 1.000 | 1.000 |  | 0.660 | 0.014 |
|  |  |  | MR_AIC_ | 1.000 | 1.000 |  | 0.628 | 0.002 |
|  |  |  | SR_top5_ | 1.000 | 1.000 |  | 0.678 | 0.020 |

**Table S2 Continued**

|  |  | Power | | | | False Positives | | |
| --- | --- | --- | --- | --- | --- | --- | --- | --- |
| p | g^2^ | No  Correction | Main Effects Correction | Additive | Co-dominant | No  Correction | Additive | Co-dominant |
|  |  |  | MB-MDR_adjust_ | 0.012 | 0 |  | 0.700 | **0.048** |
|  |  |  | MB-MDR_1D_ | 0.084 | 0.068 |  | 0.714 | 0.100 |
|  |  |  | MB-MDR_list_ | 0.078 | 0.032 |  | 0.734 | **0.074** |
|  | 0.01 | 0.154 | SR_perm_ | 0.074 | 0.04 | 0.980 | 0.682 | 0.080 |
|  |  |  | SR_0.05_ | 0.006 | 0 |  | 0.582 | 0.006 |
|  |  |  | MR_AIC_ | 0.004 | 0 |  | 0.570 | 0.006 |
|  |  |  | SR_top5_ | 0.006 | 0 |  | 0.610 | 0.016 |
|  |  |  | MB-MDR_adjust_ | 0.120 | 0.006 |  | 0.736 | **0.050** |
|  |  |  | MB-MDR_1D_ | 0.332 | 0.208 |  | 0.788 | 0.182 |
|  |  |  | MB-MDR_list_ | 0.312 | 0.062 | 0.994 | 0.790 | 0.086 |
|  | 0.02 | 0.712 | SR_perm_ | 0.248 | 0.048 |  | 0.718 | 0.096 |
|  |  |  | SR_0.05_ | 0.050 | 0 |  | 0.606 | 0.008 |
|  |  |  | MR_AIC_ | 0.052 | 0 |  | 0.594 | 0.008 |
|  |  |  | SR_top5_ | 0.062 | 0 |  | 0.620 | **0.026** |
|  |  |  | MB-MDR_adjust_ | 0.422 | 0.014 |  | 0.796 | **0.042** |
|  |  |  | MB-MDR_1D_ | 0.556 | 0.17 |  | 0.844 | 0.144 |
|  |  |  | MB-MDR_list_ | 0.534 | 0.05 |  | 0.848 | **0.040** |
| 0.5 | 0.03 | 0.970 | SR_perm_ | 0.386 | 0.008 | 1.000 | 0.786 | **0.058** |
|  |  |  | SR_0.05_ | 0.262 | 0.002 |  | 0.676 | 0.010 |
|  |  |  | MR_AIC_ | 0.266 | 0.002 |  | 0.658 | 0.008 |
|  |  |  | SR_top5_ | 0.278 | 0.002 |  | 0.688 | 0.016 |
|  |  |  | MB-MDR_adjust_ | 0.858 | 0.134 |  | 0.898 | **0.042** |
|  |  |  | MB-MDR_1D_ | 0.878 | 0.132 |  | 0.906 | **0.054** |
|  |  |  | MB-MDR_list_ | 0.878 | 0.112 |  | 0.918 | **0.032** |
|  | 0.05 | 1.000 | SR_perm_ | 0.730 | 0 | 1.000 | 0.876 | **0.054** |
|  |  |  | SR_0.05_ | 0.704 | 0.004 |  | 0.834 | 0.004 |
|  |  |  | MR_AIC_ | 0.688 | 0.004 |  | 0.810 | 0.002 |
|  |  |  | SR_top5_ | 0.720 | 0.002 |  | 0.852 | 0.018 |
|  |  |  | MB-MDR_adjust_ | 1.000 | 0.684 |  | 1.000 | **0.056** |
|  |  |  | MB-MDR_1D_ | 1.000 | 0.646 |  | 1.000 | **0.056** |
|  |  |  | MB-MDR_list_ | 1.000 | 0.646 |  | 1.000 | **0.046** |
|  | 0.1 | 1.000 | SR_perm_ | 1.000 | 0.186 | 1.000 | 0.998 | **0.050** |
|  |  |  | SR_0.05_ | 1.000 | 0.172 |  | 0.998 | 0.008 |
|  |  |  | MR_AIC_ | 1.000 | 0.146 |  | 0.998 | 0.002 |
|  |  |  | SR_top5_ | 1.000 | 0.178 |  | 0.998 | 0.032 |
